# Is there still evolution in the human population?

**DOI:** 10.1007/s42977-022-00146-z

**Published:** 2023-01-02

**Authors:** Ádám Kun

**Affiliations:** 1 Department of Plant Systematics, Ecology and Theoretical Biology, Eötvös University, Budapest, Hungary; 2 Parmenides Center for the Conceptual Foundations of Science, Pöcking, Germany; 3 Institute of Evolution, Centre for Ecological Research, Budapest, Hungary; 4 MTA-ELTE Theoretical Biology and Evolutionary Ecology Research Group, Budapest, Hungary; 5 MTA-ELTE-MTM Ecology Research Group, Budapest, Hungary

**Keywords:** Human genetics, Migration, Population structure

## Abstract

It is often claimed that humanity has stopped evolving because modern medicine erased all selection on survival. Even if that would be true, and it is not, there would be other mechanisms of evolution which could still led to changes in allelic frequencies. Here I show, by applying basic evolutionary genetics knowledge, that we expect humanity to evolve. The results from genome sequencing projects have repeatedly affirmed that there are still recent signs of selection in our genomes. I give some examples of such adaptation. Then I briefly discuss what our evolutionary future has in store for us.

## Introduction

The question whether humans are still evolving keeps resurfacing. While I have not seen any peer-reviewed publication claiming that human evolution has stopped, one can read such statements in books and hear them in interviews by prominent scientists and naturalists much too often. It is also a public belief, including among biology students. The literature only refers to this general feeling and then reaffirms that evolution has not stopped in humans (Ayala 2015). In this review, I would like to address this question so that a bit less confusion floats around in the scientific community as well as in the public.

Here I will use *change in the allele (genotype, genetic variation) frequencies of populations over time* as the definition of evolution (Futuyma and Kirkpatrick 2017; Gillespie 1998; Hall and Hallgrímsson 2008; Noor 2018). Change in frequencies of heritable traits over time is a definition (Jablonka and Lamb 2005) more in line with the extended evolutionary synthesis (Pigliucci 2007). By this latter definition, cultural evolution would also be included, but people already agree that there is plenty of cultural evolution in our species.

When it comes to evolution, one wants to see big changes, visible changes, something befitting a good movie. A club-wielding caveman with a heavy brow-ridge and no forehead versus a modern man clad in Armani is a good enough contrast. A fish crawling out to conquer land is one too. Tiny lizards becoming gigantic dinosaurs (or mutating to become Godzilla) are also fine. Bacteria evolving to be able to feed on a novel sugar source (Blount et al. 2008), some small passerine changing its beak (Grant and Grant 2002) to be able to crack bigger seeds, or a bat virus changing host is not that fantastic a story.

Our general image of mutations is also of dramatic extremes: It either produces misshapen figures like Quasimodo or superhumans like X-men. Mutants need to exhibit something truly novel: superfast regeneration, telepathy, control over metals, eyes that shoot laser beams, or spider-sense (whatever). The expectation of misshapen form is—unfortunately—well founded. The sudden appearance of superhuman abilities is less so. Beneficial mutations (as we will see later) most often manifest in minuscule changes to peptides that allow us to avoid some pathogen, slight changes to the developmental program that, for example, allows the production of lactase into adulthood, etc. Again, evolution is infuriatingly unspectacular. Or is it? It allowed us to imagine and manufacture spectacular things from the seven wonders of ancient times, through beautiful works of art to the marvels of the digital age. (Do you remember the last time you had to use transparencies for a lecture, or used snail mail to correspond with some friends or relatives?)

One also wants evolution to be fast. Fast enough to be observable in our lifetime. This can be done in a laboratory with an organism like *Escherichia coli*, *Drosophila melanogaster*, or *Caenorhabditis elegans*, but do we truly expect humans to evolve that fast? My grandparents had seen three generations of their progeny (I hope I will live long enough to see my great-grandchildren), and there are undoubtedly people who have seen four or five generations of humans. Can we expect any visible change in such a short time? Even in the laboratory, it takes more than 2–5 generations to see some effect even if very strong selection pressure is administered.

In this review, I will demonstrate that the fact that humans are still evolving can be understood by applying the knowledge of basic evolutionary genetics. Human evolution might not be as spectacular as some imagine, but it is still an ongoing process that was shaping our life, is shaping our life, and will be shaping our life as long as humans exist.

## The evolutionary genetics view of human evolution

Let us take an ideal population in which infinitely numerous members experience no mutation, no selection, they do not migrate and when they reproduce sexually, they are matched up randomly with a member of the other sex (panmixis). There is no evolution in such population. This is one of the fundamental basics of evolutionary genetics. Any population of evolutionary units which deviates from this idealized population can undergo evolution. Our task is to discern if these characteristics are true or not for humanity in 2022 (or at any other date you might read this paper). We will go through each of them individually.

### Panmixis

Panmixis means that mating is random, i.e., the probability of any two individuals of the opposite sex forming a pair is the same irrespective of their genetic makeup. Personal experience tells me that we are not paired by some matchmaking lottery, on the contrary, pair forming is very much assortative. Assortative mating can be defined as the non-random coupling of individuals on the basis of resemblance in one or more phenotypic characteristics (Buss and Barnes 1986). Spouses are similar in various traits (Price and Vandenberg 1980; Watson et al. 2004), such as ethnicity, socioeconomic status, religion, and politics. They also moderately correlate in educational attainment (Mare 1991), intellectual traits (Plomin 1999), vocational interest, and personality variables. Correlation might be low for anthropometric characteristics, but it is significant as assortative mating is also evident on the level of genetics (Conley et al. 2016, Robinson et al. 2017). Thus, the assumption of panmixis is violated.

Assortative mating (the lack of panmixis) does not necessarily change allelic frequencies, albeit genotype frequencies might change. Positive assortative mating (mating by similar individuals) increases homozygosity and thus decreases genetic diversity. Negative assortative mating (mating by dissimilar individuals), on the other hand, increases heterozygosity and thus increases genetic diversity. When non-random mating is directional in the sense that a certain trait is preferred by most people, then it can lead to sexual selection.

Mating can be assortative with respect to space, i.e., members of couples come from geographical proximity, as opposed to some random location that would be assumed under global panmixis. We often find that genetic and spatial distance correlate, even on the scale of Europe (Novembre et al. 2008). We generally find finer population structure within countries [France (Saint Pierre et al. 2020), Spain (Bycroft et al. 2019), the Netherlands (Abdellaoui et al. 2013), Ireland (Gilbert et al. 2017), and UK (Leslie et al. 2015)] that correlate with geography. Estonia is a good example. Estonia is a Baltic country with 1.3 million inhabitants spread over 45,339 km^2^. The genetic differences between Estonians and their place of origin correlate to a great degree (Nelis et al. 2009). As another example, in Mexico, the pre-Columbian population structure still prevails (Moreno-Estrada et al. 2014), even among the cosmopolitan population. This means that indigenous people moved very little in the intervening hundreds of years, which seems to be the case in South American countries as well (Chacón-Duque et al. 2018; Homburger et al. 2015).

Historical and cultural isolation can also result in barriers to gene flow. We can observe the genetic isolation of ethnic groups, like the bigoudens in Brittany (Salmon et al. 1986), the Pomaks of northern Greece (Panoutsopoulou et al. 2014), and the people in the Val Borbera valley (Italy) (Colonna et al. 2013) without geographical barrier. Language minorities in the Eastern Alps show reduced genetic diversity and genetic isolation (Capocasa et al. 2013). Papua New Guinea is also an example of cultural and linguistic differences strengthening genetic isolation. Genetic diversity is higher there than in any comparable-sized Eurasian area (Bergström et al. 2017), and some of the isolations are continuous since the settlement of the island more than 50 thousand years ago (Pedro et al. 2020). Similarly, the caste system in India genetically isolates people who otherwise live in the same place (Bamshad et al. 2001). Isolation is a cultural mandate so strong that the genetic trace of caste isolation is evident among the Pakistani in the UK (Overall 2009). Analogously, in Brazil, very little admixture is observed among people of different ancestry (Kehdy et al. 2015).

One of the consequences of assortative mating is that while any local population could be in Hardy–Weinberg equilibrium, the global human population is certainly not because of population structure (c.f. Wahlund effect). If, as evident from the above, humanity for the most part is still a collection of populations connected by very infrequent gene flow, the global census population size is a poor indication of the possible drift humanity experiences.

### Infinite population

The 8 billion humans inhabiting Earth seems to be a very large population, however, from the point of view of drift the size of populations that actually breeds matters. Drift is a change in allelic frequencies of neutral [or nearly neutral (Ohta 1992)] variants due to stochasticity in the population dynamics. A new variant could fixate in $4{N}_{\mathrm{e}}$ generations (where ${N}_{\mathrm{e}}$ is the effective population size, the size of a panmixing population that drifts to the same degree as an actual observed population). Human effective population size estimates range from a few hundred to a few hundreds of thousands (Bergström et al. 2020; Park 2011; Tenesa et al. 2007). At the higher end of those estimates, populations are large enough so that drift to fixation would take more years than the entire history of our species so far. But at the lower bound, it is just a few tens of thousands of years, and the effect of drift can be seen in a shorter period of time. The geographic, ethnic, or cultural isolates mentioned earlier have low effective population sizes. For example, some of the higher castes in the Indian district Jaunpur exhibit effective population sizes in the lower part of that range (Zerjal et al. 2007). The Pomaks of northern Greece and the Createans are isolated compared to the inland Greek population, and some variations drifted to high frequencies among them (Panoutsopoulou et al. 2014). There is an extensive fine-scale population structure in Galicia (Spain), resulting in local drift (Bycroft et al. 2019). Thus, there are still populations that can experience substantial drift over short periods of time.

Estimating drift based on census population size is challenging. Effective population sizes are always lower than census population sizes, but the factor could vary. Effective population size was found to be about half the census population size for a current cohort and a third for populations a few generations ago (Browning and Browning 2015). For Denmark, the estimated effective population size is around half-million, one-tenth of its census population size. The population seems to be quite homogenized, with only little trace of geographical origins (Athanasiadis et al. 2016).

There are two main causes of low population numbers which then makes drift stronger in a population, whose effect then can be observed hundreds of years later. One is a population bottleneck due to some catastrophic event, the second is the founding of a new population in some other geographic area. There are ample examples of the founder effect. Effective population sizes have clear minima around 12 generations ago in the Americas (Browning et al. 2018), which are caused by diseases of European origin among the indigenous people, and voluntary or involuntary migration in the case of European and African people. There are other recent examples of the founding effect on the genetic makeup of certain populations. Among the Boers of South Africa the prevalence of porphyria variegata (Dean 1968) is around 0.4% (Botha and Beighton 1983) which is many orders of magnitude higher than in other populations. Dean traced its origin back to a single couple (Dean 1968) living in the seventeenth century. Another example is the peopling of the Pingelap atoll which happened around 1775 when some suffered a shipwreck on it. There was a mutation among the survivors which causes a certain color-blindness. This condition has a prevalence of 1:20,000 in humanity as a whole, but around 10% there due to the presence of an allele in the founding population, and not much intermixing ever since (Brody et al. 1970).

Geographic and cultural assortative mating results in a fine-scaled population structure that might result in local populations experiencing drift. Such small local populations are often the result of migration, which is another characteristic of an ideal population that does not hold for humanity.

### No migration

Migration has no bearing on the overall allelic frequency of humanity on Earth, as there is no human-inhabited another planet to migrate to and from. On the local level, however, migration affected the genetic make-up of populations. Major migratory events have shaped human history. Some of them are ancient (Nielsen et al. 2017), like the peopling of Europe first by hunter-gatherers, then by early farmers, and then by steppe people. And some are recent, for example, the colonization of the Americas.

The peopling of Europe is characterized by waves of migration. The first anatomically modern humans that reached Europe have no progeny today (Fu et al. 2016). The next wave of hunter-gatherers populated Europe 37–14 thousand years ago. These early western hunter-gatherers had a lasting genetic legacy, especially in northwestern Europe (Haak et al. 2015). In the northeast, we also find traces of the early northeastern hunter-gatherers (Lazaridis et al. 2014). Then in the Upper Neolithic, around 7000 years ago, farmers migrated from the Fertile Crescent to Europe (Brandt et al. 2013; Mathieson et al. 2018). After that, in the early bronze age, steppe pastorals came to Europe (Haak et al. 2015). Present-day Europeans, for the most part, are mixtures of the descendant of these three major waves of migration.

The Americas were initially peopled from northeast Asia through Beringia. The ethnic makeup of the continent changed drastically in the last 12–17 generations following its discovery and colonization by Europeans. At present, in the USA, less than 1% of the population is native American, the rest having European (including Middle Eastern and North African) (72.4%), African (12.6%), or Asian (4.8%) ancestry. Ancestries of these populations could still be traced back to well-defined regions in Europe, Africa, and Asia (Han et al. 2017). In South America, the genetic composition aligns well with recorded history, but it can help uncover less documented migratory events such as that of converted Jews (Chacón-Duque et al. 2018) or the exact origin of the slaves (Schroeder et al. 2015). The different waves of immigration to Brazil can be inferred from the genetic makeup of the population (Kehdy et al. 2015). In the earlier colonized northern part, whites can trace their ancestry to Iberia, and the ancestors of the black population lived in central western Africa the area most of the Caribbean and North American slaves originate from. In the south, there are considerably more people of East African (Bantu from Mozambique) ancestry, and the European immigration of the nineteenth century (e.g., from Germany) can also be clearly observed.

Military expansion can also left its mark on the genetic composition of local populations. The expansion of the Mongol Empire (Zerjal et al. 2003) lefts its Y chromosomal trace across inner Asia [but see (Wei et al. 2018) who argue that it is the result of ordinary migration taking place earlier]. Muslim rule of the southern part of the Iberian Peninsula can be observed in the genomes of the locals (Bycroft et al. 2019). And ancient Rome became a truly cosmopolitan place with people from all over the empire living there (Antonio et al. 2019).

Migration is an ongoing process, and its volume is higher than previously estimated. In any given year since 1990, 0.226–0.258% of the global population migrate (Azose and Raftery 2019). That translate to 12–20 million people moving annually. This is considerable gene flow between localities.

### No mutation

Human mutation rate is estimated to be 1.29 × 10^–8^ mutations/base/generation; 4.27 × 10^–10^ mutations/base/year or roughly 70 mutations per our entire genome per generation (Jónsson et al. 2017). This figure alone, obtained by comparing the genomes of parent–offspring pairs, is enough to demonstrate that mutations happen. Around 140 million babies are born annually, which could roughly mean 9.8 billion new mutations entering the human population. Most of them are neutral or deleterious, but as the volume of novel mutations increases, the possibility of hitting on a beneficial one increases too. We are not going to see the beneficial effect anytime soon, as they go unnoticed. (People do not go to the physician if they feel too well, nor will they complain about it.)

We can also look at the human mutation rate in comparison with mutation rates observed in other animals and eukaryotes. Species can have different mutation rates for various reasons (Bromham 2009). The mutation rate in humans (in mutations/base pairs/generation) is greater than in other well-studied species, such as *D. melanogaster* (Arthropod, Animal), *C. elegans* (Nematoda, Animal), *Arabidopsis thaliana* (Plant), or *Saccharomyces cerevisiae* (Fungi) (Lynch 2010). Our per-generation mutation rates might be higher than those of other species (albeit not higher than those of our closest relative, see Table 1), but animals with longer generation times have lower substitution rates (substitutions/year) (Welch et al. 2008). And the number of mutations per base per year rates are lower for primates compared to *C. elegans* or *D. melanogaster*. While there is a considerable number of de novo mutations per generation, a generation for us is 26–30 years long (Fenner 2005; Moorjani et al. 2016; Tremblay and Vézina 2000). The neutral rate of evolution, which is driven by the mutation rate, is quite slow for humans and apes in general (Chintalapati and Moorjani 2020).

**Table 1:** Mutation rates of humans and selected primate and model species

	Mutations/base/generation	Mutations/base/year	Mutations/genome/generation	References
Human	1.29 × 10^–8^	4.27 × 10^–10^	74.4	Jónsson et al. (2017, Kong et al. (2012)
Chimpanzee (*Pan troglodytes*)	1.2 × 10^‒8^	4.6 × 10^–10^	35	Venn et al. (2014)
Western gorilla (*Gorilla gorilla*)	1.74 × 10^‒8^	9 × 10^−10^		Besenbacher et al. (2019)
Sumatran orangutan (*Pongo abelii*)	1.66 × 10^‒8^	6.64 × 10^–10^		Besenbacher et al. (2019)
Owl monkeys (*Aotus nancymaae*)	0.81 × 10^‒8^	1.23 × 10^–9^	0.502	Thomas et al. (2018)
Gray mouse lemurs (*Microcebus murinus*)	1.64 × 10^−8^	4.37 × 10^−9^	67	Campbell et al. (2021)
Mouse (*Mus musculus*)	5.4 × 10^−9^		28	Uchimura et al. (2015)
*Drosophila melanogaster*	8.4 × 10^–9^	8.4 × 10^–8^	1.99	Haag-Liautard et al. (2007)
*Caenorhabditis elegans*	1.29 × 10^–8^	2.00 × 10^–6^	2.1	Denver et al. (2004)
*Saccharomyces cerevisiae*	3.3 × 10^‒8^		4.01	Kondrashov and Kondrashov (2010, Lynch et al. (2008)
*Chlamydomonas reinhardtii*	3.23 × 10^‒10^		0.389	Ness et al. (2012)
*Arabidopsis thaliana*	7.1 × 10^‒9^	5.5 × 10^−9^	7.09	Exposito-Alonso et al. (2018, Ossowski et al. (2010)

### No selection

I left selection to be discussed last on purpose. Selection is seen as something not affecting us because there is little difference in survival. Indeed, modern medicine has greatly increased human longevity (Wang et al. 2020, 2012). The world had become a safer and healthier place for the most part (Murray et al. 2020). But there are some worrying trends: Ambient air pollution has increased and there is more danger of high temperatures (due to global warming) (Murray et al. 2020). The former is expected to increase and is a leading cause of death (Burnett et al. 2018). The latter might not have increased substantially on a global scale, but some places experience unbearable hot waves and their frequency and intensity are on the rise (Seneviratne et al. 2014). There is also an increase in drug and alcohol abuse, and sugar and red meat consumption (Murray et al. 2020); however, that probably does not exert any significant selection on humanity. All in all, from a technological point of view, there could be a considerable decrease of diseases and other ailments.

But there are huge differences in access to and affordability of health care (Peters et al. 2008) among countries and among citizens living in the same country. Consequently, longevity has not increased at the same rate for every country (Wang et al. 2020). So different survival rates still exist. The oft-repeated mantra of modern medicine freeing us from selection rest on the belief that if there is a medical solution to a problem, it will be solved for every human being suffering from said condition. The COVID-19 pandemic has demonstrated that such techno-optimism is unfounded. At the time of writing this paper in March 2021, effective vaccines have been available for months now, but still, only a small fraction of the global population has been immunized. The medical solution exists, and for billions, the remedy is not even in sight.

Most of humanity experience the same selection pressure as our ancestors for thousands of years: pathogens, lack of food, and the dangers of birth. Admittedly, there is a trend in the global causes of mortality away from infectious diseases, malnutrition, and birth-related deaths toward non-communicable causes (Lozano et al. 2012; Vos et al. 2020). But it is just a trend, even if everything goes well, we are decades if not more away from a world where everyone has access to quality healthcare. Till that time, there is still selection for genetic variants that help combat, for example, pathogens. The textbook example of heterozygote advantage of the allele variant causing sickle cell anemia if homozygous (Allison 1954) still present in millions of people as malaria still selects for its retention (Elguero et al. 2015; Piel et al. 2010). New malaria variants, however, can cause severe disease despite the protective effect of the HbS allele (Band et al. 2022). Coevolution between a host and a parasite is an ongoing selection pressure on both species.

Non-intuitively, the removal of a selection pressure can also cause evolutionary change. We only stress the beneficial effect of modern medicine on survival, but we are quiet about the evolutionary consequence. People who are cured of lethal or very deleterious hereditary diseases can procreate and thus pass on their defective genes. Muller in the 1950s (1950) argued that if modern medicine lessens the selective pressure on deleterious mutations, then they will increase in frequency. Some decades ago evolutionary biologists still openly warned the public about the danger of being too good at curing people (Ayala 1986). Recently I have not really encountered this argument, but Francisco Ayala’s colloquium paper in PNAS (Ayala 2015) is a refreshing exception. Given that humans have a long generation time, doubling the incidence of a hereditary disease might take some thousands of years, but there are quite some of these diseases, thus the burden can add up (Kondrashov 1995; Lynch 2010). On the other hand, we can hope that gene therapies can offer a remedy.

Fitness is determined by survival and the number of offspring. (Number of offspring is labeled as fecundity in population biology and fertility in human demography. I will use fecundity which is more often used in evolutionary biology.) While we often stress reproductive output when it comes to other organisms, we are obsessed with survival when it comes to our own species. There is still differential survival among humanity, even among people who have access to the wonders of modern medicine. But does that differential survival drive human evolution? Life expectancy is higher than 50 years in every country, and mostly it is higher than 60 years. Consequently, everyone can expect to live past their reproductive years. (Physiologically, men remain virile throughout their life, but most will not have new children in their 50s onward). Indirect fitness benefits can still be accrued in this period. But that is minuscule compared to selection pressure through differential fecundity.

Global population estimates for the twenty-first century (Abel et al. 2016; Ezeh et al. 2020; Kc and Lutz 2017) predict that while most parts of Earth will experience a decline in population, Africa (especially sub-Saharan Africa) could triple its population. These are the more optimistic scenarios; some projections do not show any global decline. In the last decade, estimates had to be modified upward (KC 2020). Armed conflicts and economic hardship in these areas increase the birth rate (Kebede et al. 2019). [The current pandemic is also expected to increase the birth rate in poor countries (Aassve et al. 2020).] Hardship has the exact opposite effect in middle- or higher-income countries, like Hungary: The birth rate is expected to drop further. The COVID-19 pandemic so far busted births all across the developed world. So, while some countries’ populations shrink, others still grow by 2% a year. That is clear differential fecundity, especially if we consider that survival statistics are better in the more developed countries as opposed to those experiencing high annual growth rates (Wang et al. 2020, 2012).

Differential fecundity also leads to strong selection within populations. Ayala (1986) points out that as the mean offspring number decreases the variance of offspring number becomes more important. Consequently, small differences in offspring count (now nearly 100% of the children are expected to become mature adults) can have large fitness consequences. Analyzing historical data on fecundity, age at first reproduction turns out to be a very important determinant of fitness (Helle et al. 2005; Käär et al. 1996). More so than the length of the reproductive period (Byars et al. 2010; Sanjak et al. 2018) (i.e., in a constant or growing population, if someone have the same number of children, then from a fitness point of view, it is worthwhile to have them early than late or paced out). Age at first reproduction had negligible heritability in a preindustrial society (Pettay et al. 2005) but was found to have moderate one in contemporary women (Kirk et al. 2001). Age at first reproduction has less variation in societies without contraception [e.g., Allal et al. 2004; Ramirez Rozzi 2018)], so it is mostly culturally dictated. In western societies, childbearing is more of a choice than a mandate, and beside cultural influences, genetic disposition could have larger influence. We readily find genetic variations relating to reproductive behavior and the number of children (Barban et al. 2016). The heritability of these traits changes rapidly depending on the prevailing cultural environment (Briley et al. 2015), and thus, current trend might not continue in the future. But at the moment, a high number of children, especially if they come early, offers considerable selective advantage (Byars et al. 2010; Milot et al. 2011; Sanjak et al. 2018).

I have demonstrated that the human population is far from ideal, and therefore evolution could happen in it. In the next sections, I review some examples of recent adaptations to local environments, then discuss phenotypic changes that are not the result of evolution, and lastly ponder upon what the future might hold for humanity.

## Local adaptations

Humanity belongs to one species, but since our spread out of Africa, there was not a single selective force that left a mark on all of our genomes: There is little signs of recent selective sweeps (Hernandez et al. 2011). We were already living in subdivided populations at our species’ inception (Scerri et al. 2018) and then went on to populate new continents with various habitats. The challenges (main drivers of mortality) were roughly the same as discussed earlier: pathogens, malnutrition, and birth-related problems. We can observe signs of selection related to these challenges in ancient genomes (Mathieson et al. 2015) as well as in contemporary ones (Akey 2009; Field et al. 2016; Hancock et al. 2011, 2010; Lachance et al. 2012). But the pathogens and the available food sources vary in different parts of the globe, so adaptations are mostly local (Fan et al. 2016). Here, I will give some examples of such local adaptations. They can be observed as during the last thousands of years some genetic variants rose to prominence in certain populations living in specific environments. Adaptation is an ongoing process, but it takes time before subtle changes in allelic frequencies (especially if it involves many loci) can be detected, correlated with the local environment, and then the causative link discovered.

### Climatic and environmental adaptation

The abiotic environment can exert a selective force on a species. Our species had evolved on the savannahs and then moved into temperate and artic environments, to which we were not adapted. The solution was in part genetic, but in the most part cultural. Despite cultural adaptation and niche construction (Odling-Smee et al. 2003, 2013) there are examples of genetic adaptations to local environments. Aboriginal Australians seem to be selected to withstand the desert cold (Malaspinas et al. 2016). East Asians have a mutation in the ABCC11 gene which determines earwax type and, in this case, causes it to be dry compared to the wild-type wet one (Yoshiura et al. 2006). This genetic variant was selected for in the last 2000 or so generations (Ohashi et al. 2011), most probably as an adaptation to cold environments.

High-altitude poses a peculiar challenge to humans: Every breath we take contains less oxygen compared to those in the lowlands. People adapted to lower altitudes need to let their bodies get accustomed to the thinner air, and they will still burn more calories, take more breaths, and still have lower blood oxygen levels than locals. People in the Tibetan (Simonson et al. 2010), Andean (Bigham et al. 2009), and Ethiopian (Alkorta-Aranburu et al. 2012; Scheinfeldt et al. 2012) highlands are adapted to their high-altitude homes. All three populations adapted to similar environments in different ways (Beall 2006, 2007; Bigham et al. 2010). Adaptation to high altitudes could have been quick. Recent findings in the Tibetan plateau date human settlement to 30–40 thousand year ago (Zhang et al. 2018), which is very close in time to the human migration out of Africa and toward East Asia. An archaeological find in the Andes (4355–4480 m above sea level) are dated 12.4–11.8 thousand years old (Rademaker et al. 2014), 2000 years younger than the oldest known South American site (Dillehay et al. 2008).

### Pathogen defense

While the environment and the scarcity of food were the main limiting factors in temperate and artic environments, pathogens limited the population growth of hunter-gatherers in tropical and subtropical areas (Tallavaara et al. 2018). Pathogens also exerted considerable selective force throughout history; for example, the Black Death took a heavy toll on the population of Europe. It is not surprising that selective changes are often associated with immune-related regions in our genomes (Fumagalli et al. 2011; Pagani et al. 2016). Pathogen is a catchall category and populations had to deal with their own local pathogens. For example, Europeans have some immune-associated gene changes that predate the Neolithic transition (Olalde et al. 2014), and it is probably a sign of selection for resistance against local pathogens. In some more recent examples, we know with higher confidence the kind of disease certain variants are selected for. In urban areas, the frequency of a resistance allele (SLC11A1 1729 + 55del4) is elevated, which conveys resistance to tuberculosis and leprosy (Barnes et al. 2011). Resistance to Lassa hemorrhagic fever in West Africa has been selected for (Andersen et al. 2012). The plague produced convergent evolution in central European populations of very different ancestry (Laayouni et al. 2014).

Human rhinovirus C (Lamson et al. 2006) normally causes a common cold, but in some cases, it can exacerbate childhood asthma. This only happens if the 529th position in the cadherin-related family member 3 protein is a tyrosine and not the commonly found cysteine (Bochkov et al. 2015). However, the original variant found in tetrapods is the tyrosine-bearing one (Bønnelykke et al. 2014), which is mostly replaced in human populations due to it being a risk factor (Palmenberg 2017). The original variant still has around 30% frequency in some African populations, but its prevalence is less than 5% in Asia.

Sleeping sickness caused by the unicellular eukaryote, *Trypanosoma brucei*, is a widespread disease in Africa. We all harbor a gene (*APOL1*) that helps us fight this parasite (Thomson et al. 2014). While now some of us live in areas not affected by this disease, our ancestors came from Africa, and resistance was selected for. But not all subspecies of *T. brucei* can be successfully fought with the wild-type gene (DeJesus et al. 2013; Uzureau et al. 2013). There are the variants G1 and G2 harboring two point-mutations or a deletion (respectively) which confers resistance to *T. b. rhodesiense*, but also causes kidney disease if someone only has these variants in their genome (Genovese et al. 2010). This is yet another example of heterozygote advantage (Ko et al. 2013), which would maintain polymorphism at this locus.

Emerging diseases can still exert selection. If (or should I say, when) a new deadly pathogen emerges, there might be no available medical remedy, and we could only rely on our innate and adaptive immune response.

### Dietary adaptation

The ability to digest milk (lactose) in adulthood is a prime example of adaptation to changing food availability. For mammals, milk is only available from their mother, but animal husbandry allowed us to harness the milk of other animals (cattle, horse, water buffalo, camel, sheep, and goat) as a source of nourishment. Dairy products were developed early everywhere where pastoral lifestyle was adopted (Evershed et al. 2008; Orlando 2018; Outram et al. 2012, 2009; Salque et al. 2013; Wilkin et al. 2020). In European cattle herders and later in some African populations, lactase persistence appeared and quickly rose to high frequencies (Jeong et al. 2018; Ségurel and Bon 2017). Now its frequency is around 99% in norther Europe, around 70% in central Europe, and as low as 30% in southern Europe (Storhaug et al. 2017). Not every population practicing herding and consistently consuming dairy products for the last thousands of years developed lactase persistence [for example, Mongolians Orlando 2018; Wilkin et al. 2020)]. Most probably the mutation in the *MCM6* gene allowing it (Tishkoff et al. 2007) has not appeared, and thus, lactase persistence could not be selected for.

The Neolithic revolution, i.e., agriculture presented us with a more abundant, but very one-sided source of calories: starch in the seeds of cereals. There was selection for the efficient utilization of this nutrient source. The salvia amylase gene copy number (*AMY1*) varies between 2 and 15 even within one population. As humanity transitioned to a more starch-based dietary regime, there was a selection toward a higher copy number (Perry et al. 2007). Those living on high starch diet have mostly 6 or more copies of the gene (70% of them), while only 37% of those have such a high count among people still living on a more hunter-gatherer type of diet.

Food (calories) might be abundant, maybe too abundant in developed countries, but there are still 800 million people not having access to enough calories, and two billion lacking some essential nutrients from their daily food. Unfortunately, for too many people, food (the lack of it) is still a selection pressure.

## Sometimes we see evolution where there is none

I have listed some examples of adaptations. These are the most well-known examples, but there are many more, and we will uncover even more as we unravel the effects of genetic variation. There are, however, observable phenotypic changes that are most probably not the results of evolution. Sudden phenotypic changes are often–erroneously–attributed to evolution. Evolution can be quick, but if so, it requires extremely strong selection pressure, which is seldom demonstrated.

The average height of adults has considerably increased in the last 150 years, and it is still increasing (Cole 2003; Freedman et al. 2000; Fudvoye and Parent 2017). The trend is attributed to better living conditions and access to quality food and healthcare (Cole 2003; Fudvoye and Parent 2017; Zong et al. 2015). There are signs of selection on height in the past [at least in northern European populations (Turchin et al. 2012)]; however, at the moment selection on height for men is minimal (Sanjak et al. 2018), and there is actually a selection for shorter women (Byars et al. 2010; Sanjak et al. 2018). Height is not a character that is exclusively expressed in either women or men. Thus, a stronger selection on women to be shorter compared to a weaker selection on men to be taller will have the net effect of decreased height. But now, the secular trend still dominates.

Developmental changes due to changing environments can cause phenotypic change. There was a recent report (Lucas et al. 2020) that the frequency of retaining the median artery of the forearm postnatally is increasing. The prevalence increased from approximately 10% in 1880 to 30% by the end of the twentieth century. This is a considerable increase, but I doubt it to be an evolutionary change. First, its heritability has not been confirmed. Second, such a quick increase in frequency in 120 or so years would mean very strong selection pressure. What would that pressure be? If people would be dying because of the lack of it, it would be more than an anatomical curiosity. And how could an artery in the arm that most people do not have led to more kids? There could be pleiotropic effects, I admit, but that requires further studies. Too quick changes without obvious selective pressure are always suspicious.

Short-sightedness reached very high frequencies in certain parts of the globe: 70–90% in Singapore, 30–40% in Europe but around 10% in Africa. It was not so two generations ago. Short-sightedness is more prevalent in the cities as compared to rural areas, and it is more prevalent among those working on something close (like most white-collar jobs) (Foster and Jiang 2014; Goldschmidt 2003). There are two prevailing, not mutually exclusive theories of the cause for the increased prevalence of myopia (Dolgin 2015; Foster and Jiang 2014). One posits that the developing eye accommodates the most commonly used distance, and for most kids growing up in schools that is a very close distance. The other theory says that people do not spend enough time outdoors and they lack ambient natural light. More time outdoors help reduce the development of myopia (Rose et al. 2008; Wu et al. 2013). Thus, environmental and cultural factors are responsible for the phenotypic change and not changing genes (allelic frequencies). Here I should also address the myth that short-sighted people would not have survived as hunter-gatherers. First, the traditional method of hunting is not about accurate archery but about endurance (Bramble and Lieberman 2004). Second, even in a hunter-gatherer society, the worth of a man is more than just his hunting skill: For example, good storytellers are as valuable as good hunters, and they can even be totally blind and still be an important asset for their community (Wiessner 2014). Third, the gathering part of being a hunter-gatherer does not require accurate sight at farther distances.

I have given these examples as warnings that an adaptive explanation of a trait (or change in a trait) has to be substantiated.

## *Quo vadis* humanity?

If there is evolution in our species, then we can also ask where does it lead? First of all, the immediate challenges we face are not evolutionary, and something we cannot genetically adapt for. The effect of climate change will be felt in the next decades, less than one generation from now. In a hundred years or so (a bit more than three human generations) unmitigated global warming might result in a hot Earth, in which most of Earth’s surface will be uninhabitable to humans (Steffen et al. 2018).

I do not fear for our species: as Peter Ward wrote in his book Future Evolution “for the biological life span of the planet, humanity is essentially extinction-proof” (Ward 2001). This does not mean that our civilization, our way of life is extinction-proof. Civilization could very well collapse, and then, we are back to the selective forces acting on our species for the most part of our history. If we somehow manage to avert catastrophic climate change, and Earth’s climate is stabilized at some warmer but not too warm point, then current evolutionary trends could continue, and new ones might enter the scene.

Selection leads to adaptation, and lack of selection might erase adaptation. The lack of selection or decreased selection on a variant in itself might change its frequency and thus leads to evolution. Novel mutations coupled with an exponentially growing population result in a growing number of rare variants that arose in the last 100 generations (Coventry et al. 2010; Keinan and Clark 2012). These rare variants are hard to detect, and some could be deleterious and still present in the population because selection had not enough time to weed them out.

Another controversial issue is the evolution of intelligence. There is clear evidence that education attainment as a phenotypic trait is negatively correlated with the number of children one has (Beauchamp 2016). Intelligence has a genetic foundation (Deary et al. 2009) and considerable heritability (Krapohl et al. 2014). There are claims that there is an evolutionary trend toward reduced intelligence (Meisenberg 2010), but others contest this claim by showing that genetic markers associated with intelligence do not negatively correlate with the number of children (Conley et al. 2016). The prospect of falling intelligence is worrisome.

Space travel, the colonization of our solar system, and ultimately the colonization of other solar systems will result in a new bout of human evolution. When we will “boldly go where no man has gone before,” then similarly to the peopling of the continents, the founding effect will be strong. Even if a large contingent of humans is sent, and it was calculated that a multi-generational voyage to another planet might need as many as 30,000 individuals to maintain sufficient genetic diversity to found a colony (Smith 2014), that population will not have all the genetic diversity of humanity. If we also consider that people are groupish and new colonies will be founded by ethnically and/or a geographically limited set of people, then some drift is unavoidable. Once we reach our goal, there will be a new planet and a novel environment posing new selection pressures. Humanity then will again diverge.

Evolution can also be seen as a process leading to novel species. Will humanity split into two or more species? We are one species now according to the biological species concept. And we might be a different species in millions of years, but the real question is will we ever split into two or more human species? Before we jump further into the realm of science fiction, we should take a look at the past. It is actually strange to have only one *Homo* species on Earth, as for most of the existence of our genus, there were more than one species of human present on this planet. Our own species, appearing some 200 thousand years ago (Aubert et al. 2012; McDougall et al. 2005) bears the genetic mark of introgression with other human species (Hammer et al. 2011; Prüfer et al. 2017; Vernot et al. 2016). This is not surprising, as speciation might take as much as 1–3 million years (Avise et al. 1998), and the diverging lines are hybridizing, in mammals, for 2–4 million years (Fitzpatrick 2004). So in the foreseeable future, we do not expect to see any new human species.

Humanity is still evolving, and no amount of advanced technology will stop that. As a species, we can as well adapt to a post-apocalyptic world as to one in which we live more in harmony with Mother Nature. The choice is ours. The rest is done by the blind watchmaker.

## Data Availability

Data sharing not applicable to this article as no datasets were generated or analyzed during the current study.
